# Nanoemulsion Adjuvant Augments Retinaldehyde Dehydrogenase Activity in Dendritic Cells via MyD88 Pathway

**DOI:** 10.3389/fimmu.2019.00916

**Published:** 2019-05-08

**Authors:** Mohammad Farazuddin, Rishi R. Goel, Nicholas J. Kline, Jeffrey J. Landers, Jessica J. O'Konek, James R. Baker Jr.

**Affiliations:** Mary H. Weiser Food Allergy Center, Michigan Nanotechnology Institute for Medicine and Biological Sciences, University of Michigan, Ann Arbor, MI, United States

**Keywords:** nanoemulsion, epithelial cells, dendritic cells, retinaldehyde dehydrogenase, gut homing, MyD88 pathway

## Abstract

Mucosal surfaces are the primary point of entry for many infectious agents and mucosal immune responses serve as the primary defense to these pathogens. In order to mount an effective mucosal immune response, it is important to induce T cell homing to mucosal surfaces. Conventional vaccine adjuvants induce strong systemic immunity but often fail to produce mucosal immunity. We have developed an oil-in-water nanoemulsion (NE) adjuvant that provides mucosal immunity and efficient protection against mucosal pathogens when administered as part of an intranasal vaccine. In the present study, we demonstrate that intranasal immunization with NE indirectly activates the retinaldehyde dehydrogenase (RALDH) activity in dendritic cells through epithelial cell activity leading to SIgA as well as potent cellular responses and expression of α4β7 and CCR9 gut homing receptors on T cells. Confirming these findings, *ex-vivo* stimulation of splenocytes from NE nasally immunized animals showed increase in Th1/Th17 cytokines while suppressing Th2 responses. In examining mechanisms underlying this activation NE activated RALDH via MyD88 dependent pathways in DCs but did not activate the retinoic acid receptor directly. These results suggest that RALDH immune activities can be achieved by epithelial activation without direct RAR activation, which has significant implications for understanding mucosal immunity and the design of mucosal vaccines.

## Introduction

Mucosal immunity plays an important role in defending against pathogens through both innate and adaptive immune responses (1). Vaccination of mucosal surfaces involves activating mucosal dendritic cells (APC) that then circulate throughout the lymphatic system and other tissues (2, 3). This approach has been shown to provide highly effective protective immunity for several different mucosal diseases (4, 5). Therefore, new vaccine strategies that induce strong mucosal immunity and instruct antigen primed T cells to migrate toward mucosal surfaces could be critically important for protection against mucosal infections from respiratory and sexually transmitted pathogens.

While mucosal vaccines are a promising alternative to injected vaccines, the development of safe and effective mucosal vaccines remains challenging. Part of this problem relates to a lack of safe and effective mucosal adjuvants. To address the need for mucosal adjuvants, our group has developed an oil-in-water nanoscale emulsion (NE) using surfactants and highly refined soybean oil that has demonstrated significant efficacy when administered as an intranasal adjuvant (6–9). In pre-clinical studies for a range of diseases NE induces the production of antigen-specific SIgA and demonstrates protection against multiple respiratory pathogens. Notably, the adjuvant also produces titers of antigen-specific serum IgG at levels that are similar to standard vaccines while demonstrating minimal toxicity (6–8). This suggests that nanoemulsion might be a useful adjuvant for developing mucosal vaccines.

Although we have characterized the safety and efficacy of NE-based vaccines toward a wide variety of pathogens, the mechanism of action for this adjuvant remains incompletely understood. In examining the induction of mucosal immunity, it has been discovered that metabolic intermediates of dietary vitamins play an important role in regulating mucosal immune responses (9). Of particular interest is all-trans retinoic acid (RA), a vitamin A metabolite synthesized by immune cells in the gut mucosa (10). RA has been shown to enhance T cell proliferation and up-regulate the expression of T cell gut homing receptors *in vivo* (11, 12). In addition to the imprinting of antigen specific T cells, RA also induces IgA class switching in plasma cells (13). Therefore, RA and its associated metabolic pathways are thought to be a key target in the development of effective mucosal immunity. RA is produced through an enzymatic conversion of vitamin A by retinaldehyde dehydrogenase (RALDH) (14). RALDH is expressed in dendritic cells (DCs) from gut-associated lymphoid tissues, including mesenteric lymph nodes (MLN), Peyer's patches (PP), and lamina propria (LP) (15). Thus, RALDH activity is considered a potential requirement for mucosal immune activation.

Given the gut-homing effects of RA, we hypothesized that co-formulating our NE adjuvant with RA would provide a synergistic effect and further boost immunity at mucosal surfaces. Remarkably, NE alone activated RALDH in DCs, leading to the expression of gut homing receptors by T cells. This activity was observed even in dendritic cells that lack the RAR and appeared to be mediated by epithelial cell-dendritic cell interactions. These results demonstrate a novel, non-retinoic acid mediated mechanism for the induction of mucosal immunity by NE and highlight a promising strategy for the design of new vaccines against mucosal pathogens.

## Materials and Methods

### Reagents

NE was formulated by high-speed emulsification of ultra-pure soybean oil with cetyl-pyridinium chloride, Tween 80, and ethanol in water (6). RA was purchased from Sigma (R2625) and reconstituted in di-methyl-sulphoxide (DMSO) for *in vitro* studies. NE was co-formulated with RA (NE-RA) by dissolving 2.5 mg/mL soybean oil prior to emulsification as described above. Endotoxin-free OVA was purchased from Hyglos.

### Cell Lines

Cell lines were purchased from American Type Culture Collection (ATCC) and were grown at 37°C and 5% CO_2_.

#### Epithelial Cells

We used TC-1 epithelial cell line in all the experiments. TC-1 (CRL-2785) is a murine epithelial cell line derived from C57BL/6 mouse lung. TC-1 cells were cultured in RPMI 1640 + L-glutamine (Corning) supplemented with 10% heat-inactivated FBS (HI-FBS, Gemini), 1x non-essential amino acids (Gibco), 10 mM HEPES buffer (Gibco), 100 IU penicillin, and 100 μg/mL streptomycin (Gibco).

### Primary Cells

#### Generation of Bone Marrow Derived Dendritic Cells (BMDCs)

BMDCs were prepared from wild type C57BL/6J mice. Bone marrow was aspirated from the femurs and tibias using a 27-gauge syringe. After aspirating, bone marrow cells were washed with PBS and filtered through a 70 μm cell strainer to remove any debris. Cells were then re-suspended in RPMI 1,640 supplemented with 10% HI-FBS, 1 mM sodium pyruvate, 1x non-essential amino acids, 10 mM HEPES buffer, 50 μM 2-mercaptoethanol, 100 IU penicillin, and 100 μg/mL streptomycin. Cells were cultured for 6 days with 20 ng/mL GM-CSF to induce differentiation. Cultures were verified as >95% DCs by flow cytometry for CD11c.

#### Isolation of Naïve T Cells

Naïve T cells were prepared from a single cell suspension of splenocytes using an EasySep Mouse Pan-Naïve T Cell Isolation Kit (STEMCELL Technologies) according to the manufacturers protocol and re-suspended in RPMI 1640 + L-glutamine supplemented with 5% HI-FBS, 1 mM sodium pyruvate, 1x non-essential amino acids, 50 μM 2-mercaptoethanol, 100 IU penicillin, and 100 μg/mL streptomycin. Naïve T cells were used for co-culture experiments immediately after isolation.

### Co-culture Experiments

Epithelial cells were seeded in a 12-well plate at a density of 2 × 10^4^ cells/well and incubated overnight at 37°C to achieve a monolayer at ~50% confluence. Epithelial cells were then treated with PBS/DMSO, RA, NE, or NE co-formulated with RA for 6 h. After treatment, media was aspirated from the wells, NE was washed out with PBS, and BMDCs were added to the culture at a density of 1 × 10^5^ cells/well. The co-culture was incubated overnight and naïve T cells were subsequently added to the culture at a density of 1 × 10^5^ cells/well. RALDH activity and gut homing receptor expression were determined by qRT-PCR and flow cytometry at the indicated time points.

### qRT-PCR

mRNA was isolated from samples using TriZol reagent (Invitrogen) or Quick-RNA MiniPrep kits (Zymoresearch, Irvine, CA, USA). mRNA concentration was quantified by NanoDrop, followed by cDNA synthesis using a High Capacity cDNA Reverse Transcription kit (Applied Biosystems, Foster City, CA). qRT-PCR was performed using Power SYBR green PCR master mix (Applied Biosystems, Foster City, CA). Gene expression was quantified by ΔΔCt analysis and normalized to β-actin levels within individual samples. Fold change was calculated either over the untreated controls or over unstimulated samples. For qRT-PCR, PrimeTime assays from IDT technologies were used. Primer sequences are provided in Supplementary Table 1.

### Flow Cytometry

Anti-CD3 (clone 17A2), CD16/32 (clone 93), CD11c (clone N418), CD103 (clone 2E7), α4β7 (clone DATK32), and CCR9 (clone 9B1) FACS antibodies were purchased from BD Biosciences, BioLegend, or eBioscience. RALDH activity in DCs was determined using ALDEFLOUR kit (STEMCELL Technologies). Cells were re-suspended in FACS Buffer (PBS + 0.1% BSA) and non-specific binding to Fcγ receptors was blocked with anti-CD16/32 antibody at 4°C for 10 min. Cells were then stained with anti-CD11c or anti-CD103 antibodies and incubated with ALDEFLUOR reagent according to the manufacturers protocol. Expression of gut homing receptors on T cells was also determined by flow cytometry. Cells were blocked with anti-CD16/32 antibody for 10 min on ice and stained with anti-CD3, α4β7, and/or CCR9 antibodies at 4°C for 30 min. All samples were acquired on an Accuri C6 flow cytometer (BD Biosciences) and analyzed using FlowJo software.

### Animal Studies

Specific pathogen-free C57BL/6J mice were purchased from The Jackson Laboratory (female, 4–6 weeks old). All animal work was done under protocols approved by the University of Michigan Committee on the Use and Care of Animals. For *in vivo* experiments, NE vaccine was prepared by mixing 20% stock NE adjuvant with 10 μg of ova antigen per dose. Mice were immunized intranasally (*i.n*.) by pipetting 6 μl of NE or NE-RA vaccine into each nare under anesthesia (isoflurane). Ova antigen mixed with PBS or RA vehicle served as controls. Blood was collected from animals at regular intervals by saphenous vein puncture. Serum was then separated by centrifugation. At the time of euthanasia, spleen, MLN and CLN were harvested. Tissues were mechanically disrupted to release cells. Single cell suspensions were then prepared for analysis by qRT-PCR and flow cytometry. Terminal blood samples were also collected by cardiac puncture. In addition, a bronchoalveolar lavage (BAL) was performed by injecting 1 ml of PBS containing protease inhibitor cocktail. The collected fluid from the lungs was used to determine IgA.

### ELISA

Serum antibody levels were determined by enzyme linked immunosorbent assay (ELISA). Microtiter plates (NUNC) were coated with 5 μg/ml ova in 100 μL sodium bicarbonate buffer and incubated overnight at 4°C. Coating solution was then removed and plates were blocked with PBS containing 1% milk for 30 min at 37°C. After incubation, blocking solution was removed and diluted serum or BAL samples were added to individual wells for 1 h at 37°C. Plates were subsequently washed three times with ELISA wash buffer (PBS + 0.05% Tween 20) and incubated for 1 h with anti-mouse IgG or IgA antibodies conjugated to alkaline phosphatase (AP). Plates were then washed three times and incubated with Sigma Fast AP substrate (Sigma) to generate a colorimetric product. Optical density (OD) measured at 405 nm using a Spectra Max 340 ELISA reader (Molecular Devices). A cutoff value was determined by calculating OD+2^*^SD (standard deviation) from control samples. Antibody titers were defined as the reciprocal value of the highest sample dilution with an OD above the cutoff value.

### Statistics

Data were plotted using Graph Pad Prism software and are represented as mean values +/− standard error of the mean (SEM). Statistically significant differences using Two-way-ANOVA (Tukey's multiple comparisons test) and One-way ANOVA (Kruskal-Wallis multiple comparisons test) §*p* < 0.0001, #*p* < 0.0005, ^**^p < 0.005, ^*^p < 0.05. A *p* < 0.05 was considered as significant.

## Results

### Nanoemulsion Adjuvant Induces Retinaldehyde Dehydrogenase Activity by Dendritic Cells in a Co-culture Model

**Figure 1:**
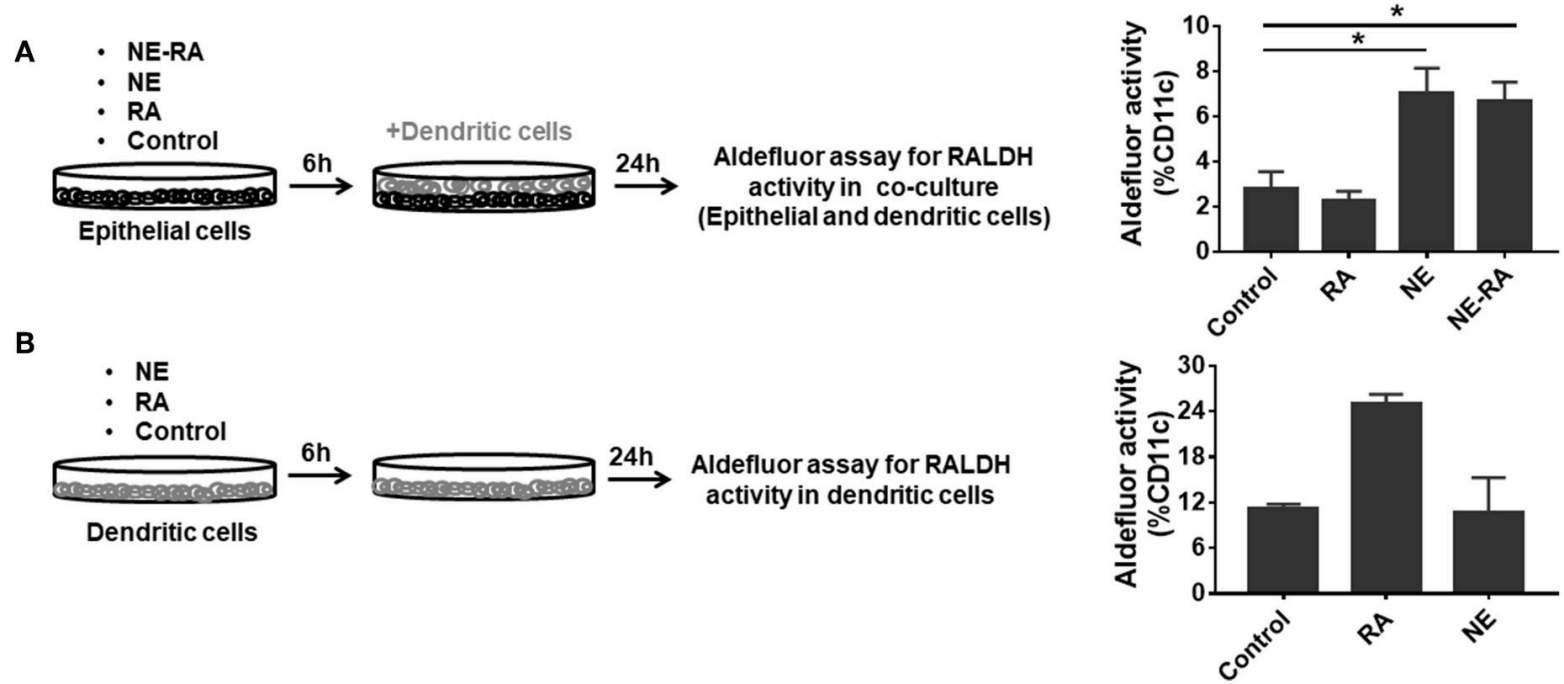
Nanoemulsion adjuvant treated epithelial cells indirectly activate retinaldehyde dehydrogenase activity in co-cultured dendritic cells. **(A)** Monolayers of epithelial cells were incubated for 6 h with the indicated treatment conditions and washed. BMDCs were then added at 1 × 10^6^ per well-seeding density. After 24 h incubation, co-cultures were stained for CD11c and aldefluor assay to examine RALDH activity. **(B)** In second set of experiment, BMDCs (1 × 10^6^) were directly treated with NE. After 6 h incubation, NE was washed off from the culture. RALDH activity was measured in BMDCs after 24 h. Representative mean ± s.e.m of two independent experiments. Statistically significant differences using One-way ANOVA (Kruskal-Wallis multiple comparisons test) ^*^p < 0.05 compared to untreated control group.

RALDH expressing CD103^+^ DCs play diverse functions in maintaining immune homeostasis. These cells induce gut homing on T cells as well as differentiation of IgA secreting plasma cells (13). We attempted to augment the mucosal adjuvant activities of the NE adjuvant by adding RA to the nanoemulsion (NE-RA). We used a co-culture model that exposed epithelial cells to NE-RA for 6 h followed by washing the residual NE-RA and co-culturing them with DCs for 24 h. We measured the RALDH activity in these co-cultures (Figure 1A). Importantly, this co-culture model replicates the microenvironment of mucosal immunization *in vivo*, where the NE vaccine interacts directly with the nasal epithelia that then leads to the activation of a network of immune cells.

As a preliminary experiment, we examined the effects of different concentrations of RA co-formulated with NE and examined RALDH activity in co-cultured DCs (JAWS II). NE-RA induced a significant increase in RALDH activity with 1 μM RA (Data not shown). We performed the same experiment with co-cultured BMDCs with NE-RA (1 μM RA). Samples were stained with an anti-CD11c antibody and incubated with a fluorescent RALDH substrate to assess enzyme activity. Surprisingly, the treatment of epithelial cells with NE alone induced significant RALDH activity in co-cultured BMDCs (Figure 1A) at levels comparable to NE-RA. Moreover, this increase in RALDH activity was restricted to CD11c^+^ cells, indicating that NE induced RALDH activity in DCs indirectly through interaction with epithelial cells. RA alone did not induce RALDH activity, potentially because the RA did not come in direct contact with DCs in the co-culture model. To determine whether NE induces RALDH activity through direct stimulation of BMDCs or if this activity is mediated through epithelial cells, BMDCs were stimulated directly with NE in the absence of epithelial cells (Figure 1B). No effects of NE on RALDH activity were observed (data not shown) in BMDCs, demonstrating that these activities are mediated through epithelial cells.

Further, to study if the observed RALDH induction was dependent upon physical contact between epithelial and BMDCs, co-cultures were performed in transwell plates in which the BMDCs were cultured in the bottom chamber of the transwell with the NE treated epithelial cells in the top chamber. We found that NE was still able to increase RALDH activity and thus, this process is not contact dependent. Together, these results suggest that NE adjuvant augments RALDH activity in BMDCs indirectly by activating epithelial cells.

### Nanoemulsion Adjuvant Increases the Expression of Gut Homing Receptors on T Cells in a Co-culture Model

**Figure 2:**
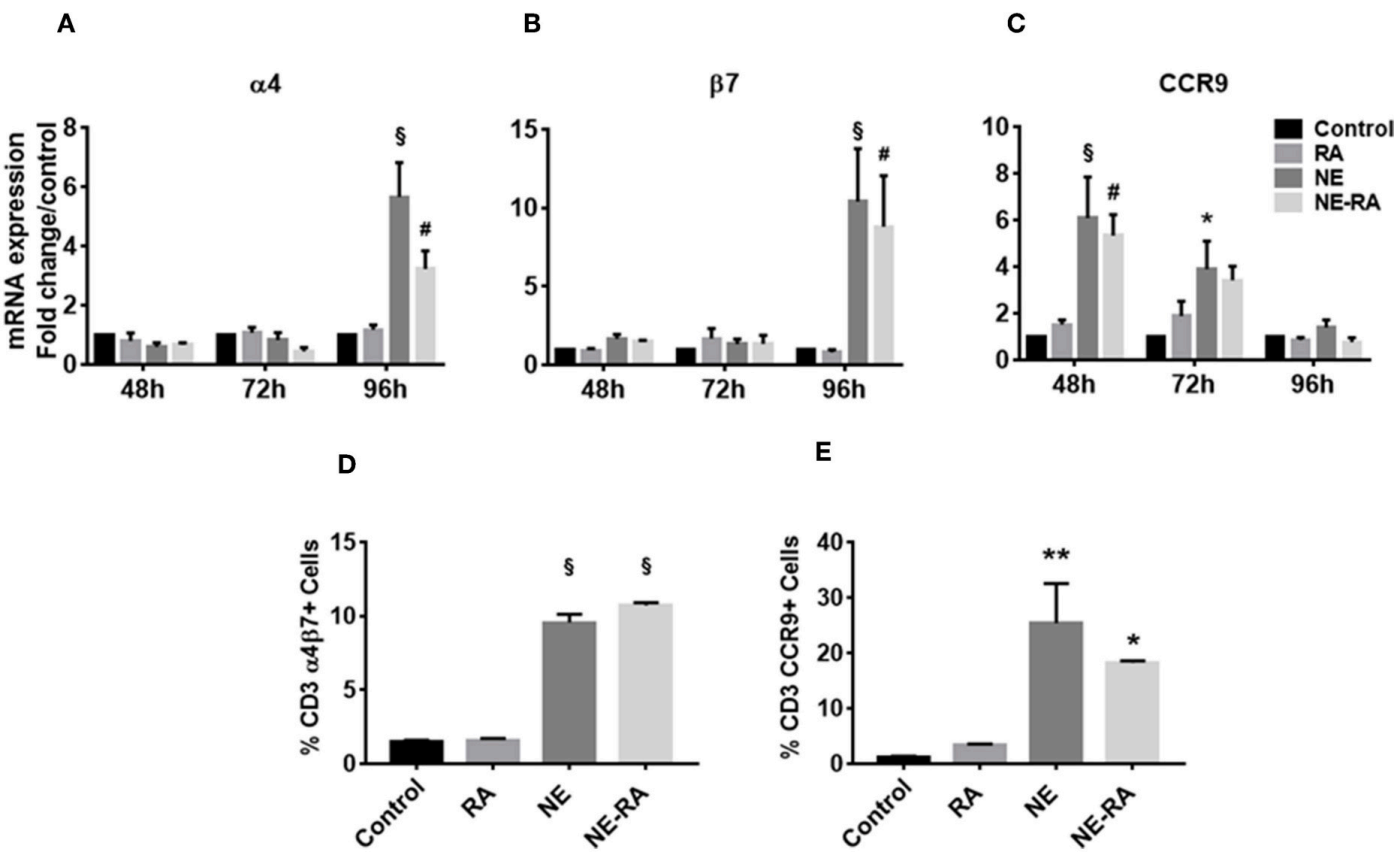
Nanoemulsion adjuvant increases expression of gut homing receptors on naïve T cells *in vitro*. NE pretreated epithelial cells were co-cultured with BMDCs for 16 h. Naïve T cells were added to co-cultures to study expression of α4 **(A)**, β7 **(B)**, and CCR9 **(C)**. mRNA was isolated from primary T cells after 48, 72, and 96 h in co-cultures. Gene expression was normalized to β-actin and fold change was calculated over the untreated control. Expression of gut homing receptors on naïve T cells was also analyzed by flow cytometry after 5 days in co-culture. Samples were stained with anti-CD3 and anti-α4β7 **(D)** or anti-CCR9 **(E)** antibodies. Representative mean ± s.e.m from two independent experiments, each performed in triplicate **(D**,**E)**. Statistically significant differences using Two-way-ANOVA (Tukey's multiple comparisons test) and One-way ANOVA (Kruskal-Wallis multiple comparisons test)^§^*p* < 0.0001 #*p* < 0.0005, ^**^p < 0.005, ^*^p < 0.05 compared to control group.

Given the NE-induced increase in RALDH activity observed in BMDCs, we sought to investigate the effects of this increase on the gut homing properties of T cells. NE pretreated epithelial cells were co-cultured with BMDCs for 24 h. Naïve, syngeneic T cells were then added to the co-culture and analyzed for up-regulation of gut homing markers α4β7 and CCR9, by qRT-PCR after 3 days and flow cytometry after 5 days. Notably, BMDCs activated by NE treated epithelial cells induced significant expression of α4 and β7 transcripts in T cells after 96 h, while the CCR9 transcript increased significantly in T cells after 48 h (Figures 2A–C). This was in agreement with previous reports indicating that expression of the gut homing receptor CCR9 is an important factor in the development of α/β integrin on T cells (16). NE also significantly increased surface expression of α4β7 and CCR9 markers on T cells after 5 days in co-culture (Figures 2D,E). These data provide additional evidence that NE induces RALDH activity in BMDCs that leads to mucosal immune activation, including the up-regulation of gut homing receptors on T cells.

### Immunization With Nanoemulsion Increases Dendritic Cell Numbers, Enhances Retinaldehyde Dehydrogenase Activity and Imprints gut Homing Markers on Mesenteric Lymph Node Cells

**Figure 3:**
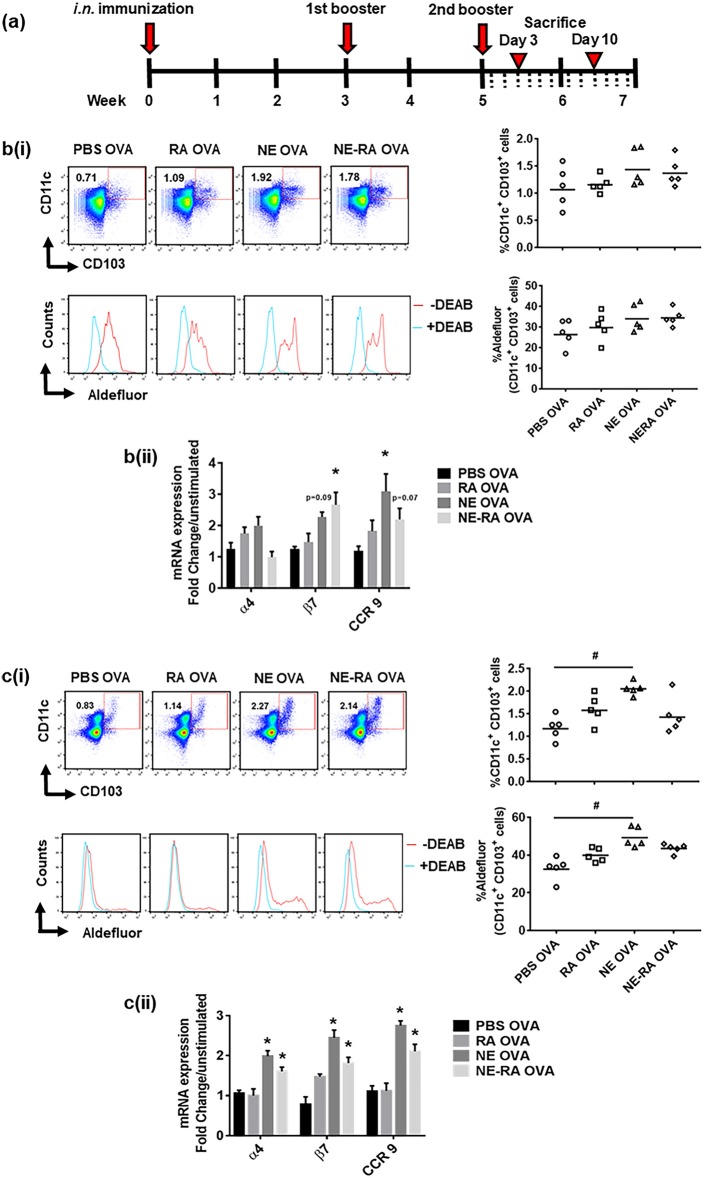
Immunization with nanoemulsion vaccine increases retinaldehyde dehydrogenase activity in dendritic cells and induces expression of gut homing receptors in lymph node cells. **(a)** Intra-nasal (i.n.) immunization schedule for *in vivo* experiments. Kinetics of RALDH activity on CD11c+CD103+ cells and expression of gut homing marker's expression in ex vivo stimulated cells in CLNs on day 3 **(b)** and in MLNs on day 10 **(c)** in immunized animals. Data is representative mean ± s.e.m. of two independent experiments. Statistically significant differences using One-way ANOVA (Kruskal-Wallis multiple comparisons test) and Two-way-ANOVA (Tukey's multiple comparisons test) #*p* < 0.0005, ^*^p < 0.05 compared to PBS OVA immunized animals.

We next sought to determine whether the increase in RALDH activity that we observed in DC cultures also translated into a similar phenotype *in vivo* in NE-vaccinated animals. Mice were immunized at week 0 and boosted at weeks 3 and 5, then euthanized 3 and 10 days after the third immunization (Figure 3a). We examined the mucosal DCs and their RALDH activity in CLNs and MLNs on both day 3 and day 10 after second immunization, as well as characterizing the effect of NE on gut homing markers of T cells in *ex vivo* stimulated cells from these lymph nodes. These studies show an increase in RALDH activity in CD11c^+^CD103^+^ cells and the gut homing markers expression in CLNs on day 3 while no changes were observed in MLNs at this time point (Figure 3b). In contrast, on day 10 in MLNs the frequency of DCs frequency and their RALDH activity was significantly enhanced. There was also a significant induction in gut homing markers in stimulated MLN cells at this time point (Figure 3c). Based on the kinetics of RALDH activity in DCs and gut homing markers expression in lymph nodes, these data suggest that NE induces RALDH activity in local draining lymph nodes that subsequently generalized to other mucosal DCs, and this subsequently enhances the expression of gut homing markers (α4, β7, and CCR9) in these cells.

### Mucosal Immunization With Nanoemulsion Produces Strong Antibody and Cytokine Responses

**Figure 4:**
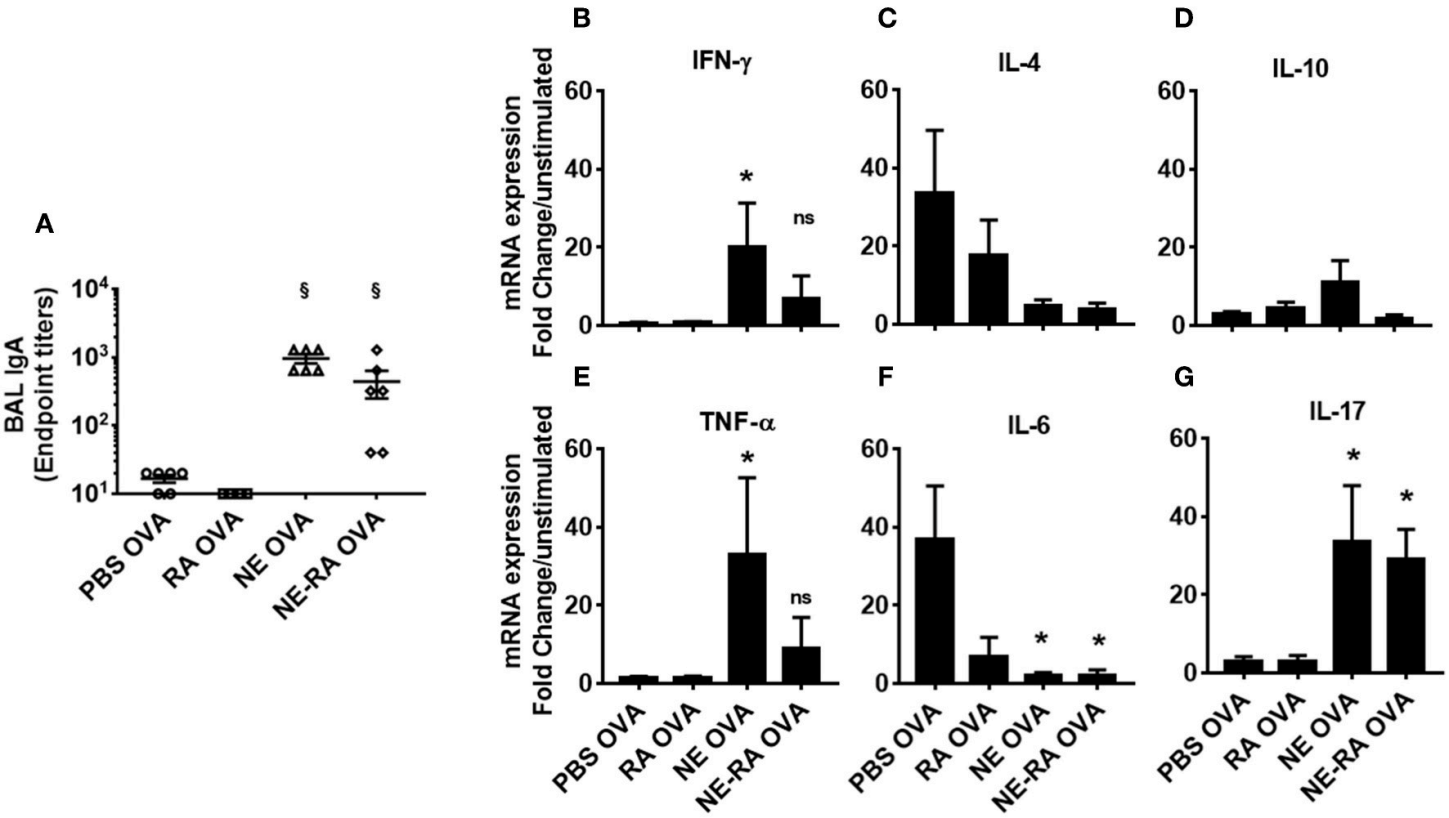
Mucosal immunization with nanoemulsion vaccine induces OVA-specific sIgA and cytokine response. **(A)** mucosal IgA levels determined by ELISA at the time of euthanasia. **(B–G)** Splenocytes isolated from immunized animals were stimulated *ex vivo* with 200 μl OVA (20 μg/mL). mRNA was collected after 24 h in culture. Gene expression was normalized to β-actin and fold change was calculated over unstimulated cells for every immunization group. Data is representative of two experiments mean ± s.e.m. Statistically significant differences using One-way ANOVA (Kruskal-Wallis multiple comparisons test) ^*^p < 0.05, ^§^*p* < 0.0001 compared to PBS OVA immunized animals.

Given the effects of RA alone in modulation of Th1 and Th2 responses (17), we investigated if co-formulation of NE and RA will have different immune response upon nasal immunization *in vivo*. The result of immunization was evaluated by OVA specific IgA antibody titers in BAL, and for cellular responses, cytokine expression was studied from *ex vivo* OVA stimulated splenocytes. Consistent with our previous studies, intranasal immunization with NE-OVA had significant increased levels of antigen specific IgA in BAL (Figure 4A). There were no significant differences in antibody production between mice that were immunized with NE vs. NE-RA, suggesting that RA did not provide additional adjuvant activity. NE induced robust cellular immune responses with increased expression of IFN-γ, TNF-α, IL-10, and IL-17 and decreased the expression of IL-4 and IL-6 from stimulated splenocytes (Figures 4B–G). Of interest, immunization with NE-RA decreased the expression of IFN-γ and TNF-α compared to NE alone. We observed similar cytokine responses when CLN cells were stimulated with the antigen (Supplementary Figure 1).

### Nanoemulsion Does Not Induce Retinaldehyde Dehydrogenase Activity in Co-cultured Dendritic Cells Either Through Retinoic Acid or TLR 2/4 Signaling

**Figure 5:**
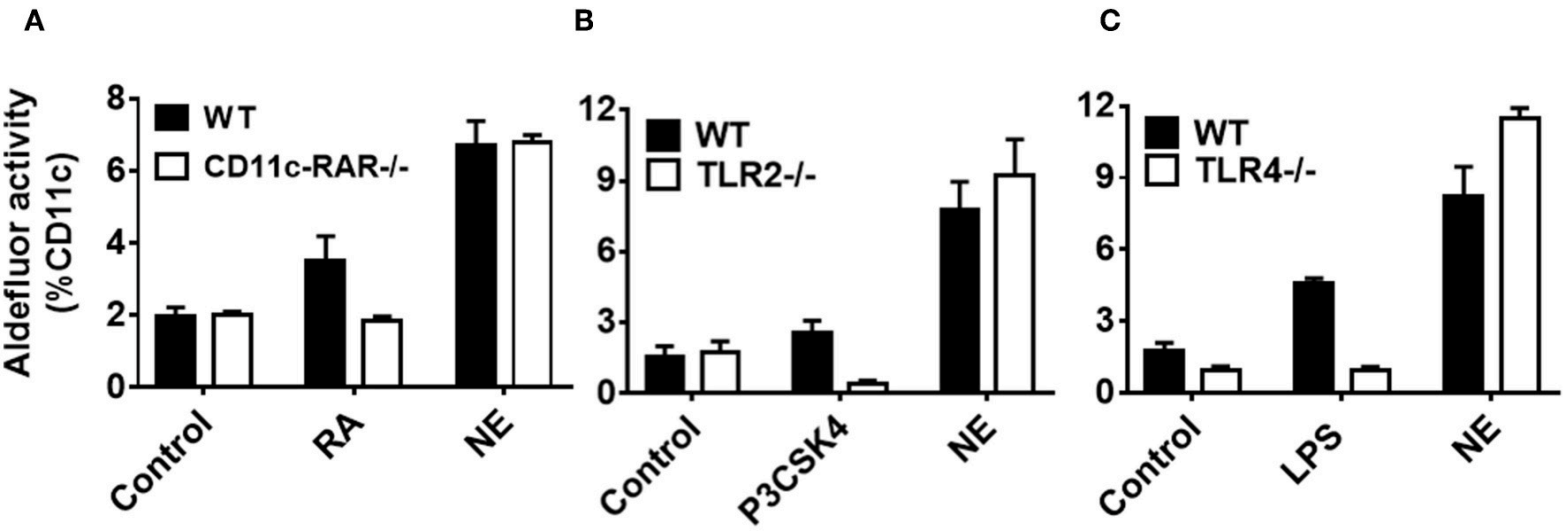
Nanoemulsion induces retinaldehyde dehydrogenase activity independent of retinoic acid, TLR 2 or TLR 4 signaling in co-cultured dendritic cells. NE treated epithelial cells were co-cultured with BMDCs isolated from **(A)** CD11c-RAR^−/−^, **(B)** TLR2^−/−^, and **(C)** TLR4^−/−^ animals. Co-cultures were washed and stained for CD11c and RALDH activity with Aldefluor kit. Representative mean ± s.e.m of two independent experiments performed in technical triplicates.

Having established NE induces RALDH immune activation in DCs and the gut homing phenotype on T cells, we sought to investigate the mechanism. To date, multiple factors including RA and certain TLRs have been shown to induce RALDH activity in DCs (16, 18, 19). We have previously shown that NE can activate TLR2 and TLR4 in TLR reporter cell lines through receptor aggregation, and have demonstrated that NE adjuvant activity is partially mediated through these pathways *in vivo* (20). Given this, we hypothesized that the NE treatment of epithelial cells might be indirectly triggering RALDH activation in co-cultured BMDCs either by RA mediated signaling or by activating TLR-2 or TLR-4. To test this, NE pretreated epithelial cells were co-cultured with BMDCs from WT and CD11c-RAR^−/−^ mouse. We found that RA deletion in DCs had no effect on RALDH activity and NE had additional ways of activating the enzyme (Figure 5A).

To investigate other signaling pathways, we isolated BMDCs from either TLR-2^−/−^ or TLR-4^−/−^ animals, and co-cultured them with epithelial cells pretreated with NE. Of interest, there was no significant difference in RALDH activity between WT and TLR-2^−/−^ or TLR-4^−/−^ BMDCs (Figures 5B,C), suggesting the involvement of multiple pathways in RALDH activation. Taken together, these results indicate that NE can activate the RALDH enzyme independent of RA signaling, potentially through multiple pathways.

### Nanoemulsion Activates Multiple Innate Receptors Leading to Increased Retinaldehyde Dehydrogenase Activity

**Figure 6:**
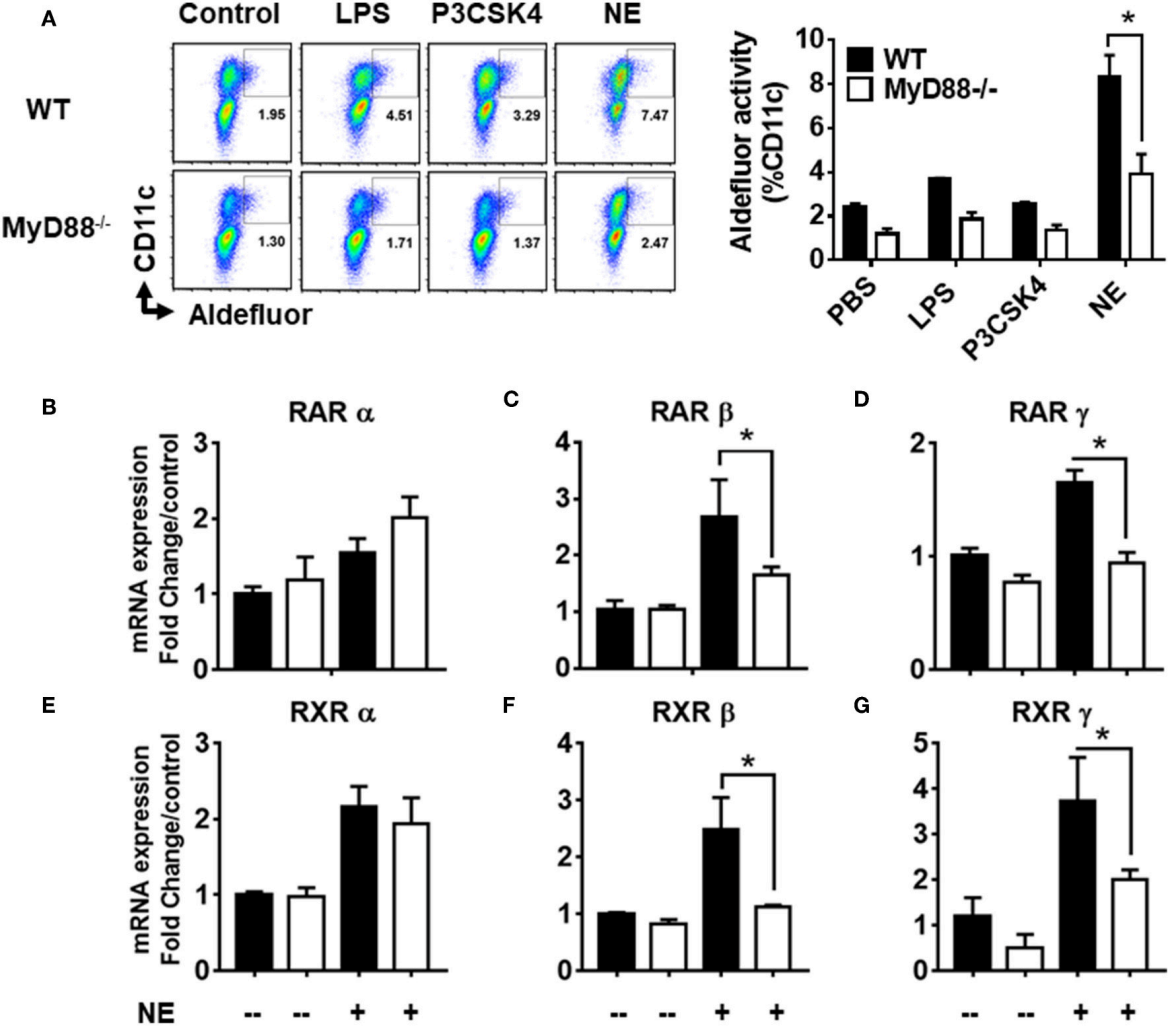
Nanoemulsion mediates retinaldehyde dehydrogenase activity in dendritic cells *via* MyD88 pathway. Nanoemulsion treated epithelial cells were co-cultured with BMDCs from WT and MyD88^−/−^ animals. RALDH activity was measured in CD11c cells with aldefluor kit after 24 h. **(A)** Representative data of experiments performed in technical duplicates. **(B–G)** Co-cultures were harvested after 16 h and CD11c cells were separated using CD11c positive isolation kit (Stem cell technologies). Gene expression was analyzed by qRT-PCR. β-actin was used as a housekeeping gene and fold change was calculated over the WT untreated control. Data presented here is mean ± s.e.m. pool of two independent experiments done in technical duplicates. Statistically significant differences using One-way ANOVA (Kruskal-Wallis multiple comparisons test) ^*^p < 0.05 compared to untreated control group.

Given that NE did not appear to work through TLR2 or 4 receptors, we sought to determine whether the activity was mediated through MyD88, as it is the common adaptor protein shared by several different families of innate receptors. We co-cultured BMDCs from MyD88^−/−^ and WT mice with NE treated epithelial cells and measured RALDH activity by aldefluor assay. While WT BMDCs showed a significant increase in RALDH activity when co-cultured with NE treated epithelial cells, the activity was abrogated significantly in MyD88^−/−^ BMDCs (Figure 6A). To gain further insight into NE mediated RALDH activity, we analyzed the expression of RAR and RXR isoforms from NE treated WT and MyD88^−/−^ BMDCs. Our results showed a significant decrease in RAR and RXR mRNA (β and γ isoforms) in the treated MyD88^−/−^ DCs, while there was no change in the α isoform (Figures 6B–G). Taken together our data suggested that NE treated epithelial cells activate multiple, as yet unidentified innate pathways in dendritic cells and utilizes the MyD88 pathway to enhance RALDH activity.

## Discussion

The development of safe and effective mucosal vaccines remains an open challenge in the field of immunology. Recently, it has been discovered that RA, a key metabolite in the vitamin A pathway, plays an important role in the development of mucosal immune responses. RA is naturally produced from Vitamin A by RALDH and has been shown to increase the expression of α4β7-integrins and chemokine receptor CCR9, thereby directing T cell migration to the gut mucosa (12). Therefore, an adjuvant capable of enhancing RALDH activity in mucosal DCs would be a promising candidate for boosting immunity at mucosal surfaces. Previous work in our lab has demonstrated that NE, a nasal vaccine adjuvant, induces strong mucosal and systemic immune responses including a balanced Th1/Th2/Th17 cellular response, along with the production of sIgA at mucosal sites (6, 8, 21). We hypothesized that co-formulating NE with RA might enhance the mucosal responses by improving mucosal homing of antigen-specific T cells. Unexpectedly, NE alone increased RALDH activity in the absence of any RAR ligand, suggesting that this may be a unique mechanism of its adjuvant activity.

Our data unequivocally demonstrate NE induction of RALDH and activation of its immune activities through non-receptor mediated pathways. Our *in vitro* co-culture studies showed that epithelial cells pre-treated with NE-RA induced dose dependent RALDH activity in co-cultured DCs that peaked at the 1 μM concentration (Data not shown). While the treatment of epithelial cells with RA alone did not induce any RALDH activity in co-cultured BMDCs, epithelial cells treated with NE were capable of inducing activity in the BMDCs *in vitro*. In contrast to RAR ligand, direct treatment of BMDCs with NE had no effect on RALDH activity. This indicated that NE induction of RALDH activity on BMDCs is mediated indirectly through epithelial cells. These results were in accordance with our previous findings demonstrating that *i.n*. immunization with NE-GFP introduces NE into nasal epithelial cells and these cells indirectly transfer it to the dendritic cells (22). Since gut-specific T cells migrate to mucosal surfaces based on the expression of homing receptors under the influence of RA (12), we also examined the expression of gut homing receptors (α4β7 and CCR9) on lymphocytes. We found a significant increase of α4β7 and CCR9 expression on T cells when cultured with NE pretreated epithelial cells and BMDCs *in vitro*. Importantly, these studies established that NE treatment, in the absence of RA, is capable of enhancing gut homing on co-cultured T cells. Thus, these *in vitro* studies indicated NE induced both RA generating enzymes and the target proteins of RA treatment, and could induce this activity through epithelial cells.

We confirmed the *in vitro* observations employing *in vivo* studies in animals. Immunization with NE resulted in an increase in the CD11c^+^ CD103^+^ DC population with significantly increased RALDH activity. This study shows that changes in DCs and gut homing expression in MLNs occur subsequent to changes in CLNs. To further clarify this progression, we evaluated mucosal DCs for RALDH activity and gut homing marker expression in cervical and mesenteric lymph nodes (CLNs and MLNs) at days 3 and 10 post immunization. On day 3 in CLNs there was increased RALDH activity in DCs and increased gut homing marker expression in NE-treated animals. Also on day 3, we observed an increase in antigen-specific IgA in the BALF. In addition to inducing RALDH activity and gut homing on T cells, RA has been shown to promote differentiation of IgA secreting plasma cells *in vitro* while also inducing antigen specific sIgA *in vivo* (13). Despite these changes in CLNs at this early time point, we did not observe any changes in MLNs. In contrast, on day 10 MLNs DCs were increased in number and had greater RALDH activity, along with higher gut homing marker expression. These findings indicate that the increase in RALDH activity occurs in DCs in CLNs at the day 3-time point, followed by an increase in MLN at day 10.

Taken together, these findings suggest that NE treatment of epithelial cells confers DCs with increased RALDH activity, resulting in lymphocyte homing to the gut and a significant increase in antigen specific mucosal sIgA production. In previous work by our group, stimulation of TLR HEK clones with NE significantly activated TLR2 and TLR4 (20). In that study NE immunization of MyD88^−/−^, TLR2^−/−^, TLR4^−/−^, or WT animals produced similar IgG titers, suggesting that activation of the TLR-MyD88 innate immunity pathway in DC is not required for IgG production. In contrast, lymphocytes in the spleens of MyD88^−/−^, TLR2^−/−^, and TLR4^−/−^ animals had increased Th2 (IL5 and IL13) and decreased Th1 cytokine production (IFN-y) as compared to those in WT animals. Along with the current work, these data indicate the NE-induced antibody response is independent of MyD88 but the induction of Th1 immune polarization is MyD88 dependent (20). This suggests that RALDH activation via MyD88 pathway may play significant role in polarization of immune responses by NE adjuvant activity.

Of interest, NE and RA do not produce identical immune stimulation, suggesting multiple pathways are involved for NE. It has been well documented that RA can dampen Th-1 and Th-17 immune responses and induces expansion of Treg cells (23, 24). In contrast, NE has been shown to induce Th-1/Th-17 immunity while suppressing Th2 responses (20, 21). Cytokine data suggested the NE immunized animals had a significant increase in Th1 cytokines that was dampened in NE-RA immunized animals; however, there was no difference between these groups in Th2 (IL-4 and IL-6) and Th17 (IL-17) cytokine production. These results indicate that NE alone is sufficient in inducing balanced Th1/Th2 immunity, and RA may block some effector functions and Th17 of NE through regulatory pathways.

It is well-established that IL-10 plays a crucial role in providing protection against infections, generating cancer immunity and inducing immune tolerance in Th-2 associated allergic diseases (25). IL-10 dampens Th2 responses, increases CD8 T cell mediated immunity and provides a balanced Th1/Th2 response to combat pathogens (26). Notably, our prior studies have demonstrated that the NE adjuvant is capable of inducing both IL-10 and Tregs, modulating existing Th2 skewed immune responses toward a more balanced immune phenotype (21, 27). In agreement with our previous studies, our data demonstrate that mice immunized with NE alone produced increased levels of IL-10 as compared to PBS controls (21). However, the mechanism by which NE induces IL-10 production in immunized animals was unknown. Interestingly, our observation of increased IL-10 expression in NE immunized mice corresponds well with other reports that vitamin A metabolites induce IL-10 producing Tregs (28). Together, the induction of IL-10 by NE lends further support to our conclusion that NE activates the RALDH in DCs and identifies potential applications for NE as an immunomodulatory agent.

To understand the mechanism by which NE treatment induced RALDH activity, we co-cultured BMDCs isolated from CD11c-RAR^−/−^ animals. We observed NE treated epithelial cells were capable of stimulating RALDH activity in RAR defective BMDCs, while RA was ineffective in these cells. This suggested that NE activates RALDH in BMDCs independently of RA signaling. To further investigate the mechanism of this activation several signaling pathways were considered. There is growing consensus that intestinal epithelial cells have the ability to stimulate RA synthesis in co-cultured DCs (29). In addition to RA, several other factors have been documented to induce *aldh1a2* transcripts in antigen presenting cells, including TLR ligands (14, 16, 25, 30). Previously, we have shown that the NE adjuvant activates both TLR2 and TLR4 in TLR reporter cells (20). Notably, some of the studies have described the critical role of TLR2 and TLR4 in the induction of RALDH activity in DCs (18, 19, 31). To investigate the role of TLR ligands, we performed co-culture experiments with TLR2^−/−^ and TLR4^−/−^ BMDCs. RALDH activity was not decreased when NE treated epithelial cells were co-cultured with TLR2^−/−^ or TLR4^−/−^ BMDCs, indicating neither TLR2 or TLR4 were involved in RALDH activity. Therefore, we targeted the downstream adaptor protein MyD88 that is involved in the signaling of many of innate receptors. MyD88 deletion reduced NE mediated RALDH activity significantly, indicating the involvement of MyD88 pathway and the possible involvement of other innate signaling pathways. Thus, while NE does not regulate RALDH activity in DCs via TLR2, TLR4, or RAR, the involvement of MyD88 suggests involvement of other innate receptors.

In summary, we have identified a unique mechanism by which NE adjuvant induces RALDH activity to produce mucosal immunity in gut associated lymphoid tissues. In contrast other vaccination strategies targeting the RA pathway have produced similar gut homing effects, but have failed to generate significant systemic immune responses (32). The unique ability of the NE to produce both mucosal and systemic immunity highlights its potential as an adjuvant for mucosal pathogens.

## Data Availability

The raw data supporting the conclusions of this manuscript will be made available by the authors, without undue reservation, to any qualified researcher.

## Ethics Statement

All animal work was done under protocols approved by the University of Michigan Committee on the Use and Care of Animals.

## Author Contributions

MF and JB designed the studies, executed the experiments and prepared the manuscript. JO and JL helped in immunizing, bleeding, and sacrifice of the animals and gave input to the studies. RG standardized the protocol for bone marrow derived dendritic cells isolation. NK helped with qPCR from *in vitro* experiments.

### Conflict of Interest Statement

JB owns stock in a company, Nanobio, that has licensed the nanoemulsion technology described in this paper. The remaining authors declare that the research was conducted in the absence of any commercial or financial relationships that could be construed as a potential conflict of interest.

## Supplementary Material

Supplementary Figure 1 Mucosal immunization with nanoemulsion vaccine induces similar cytokine responses in stimulated CLN cells. **(A–D)** Single cell suspension from (CLNs), isolated from immunized animals were stimulated *ex vivo* with 200 μl OVA (20 μg/mL). mRNA was collected after 24 h in culture. Gene expression was normalized to β-actin and fold change was calculated over unstimulated cells for every immunization group. Representative data is mean ± s.e.m of two independent experiments. Statistically significant differences using One-way ANOVA (Kruskal-Wallis multiple comparisons test) ^*^*p* < 0.05 compared to untreated control group.

Supplementary Table 1 List of all primer sequences used in the manuscript.
