# Supplementary figures and images for: Nanoemulsion Adjuvant Augments Retinaldehyde Dehydrogenase Activity in Dendritic Cells via MyD88 Pathway
Source: Front Immunol. 2019 May 8;10:916. doi: 10.3389/fimmu.2019.00916 (PMC6517504; doi:10.3389/fimmu.2019.00916)

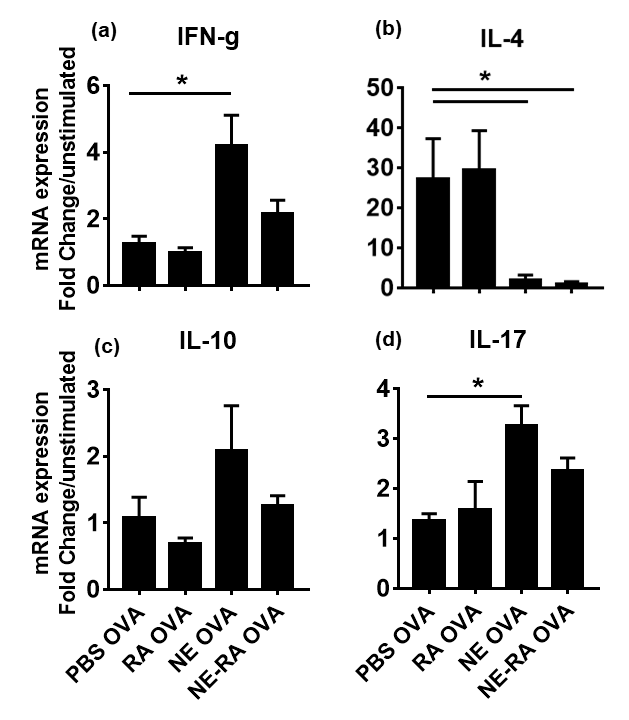

Supplement: Supplementary Figure 1 — Mucosal immunization with nanoemulsion vaccine induces similar cytokine responses in stimulated CLN cells. (A–D) Single cell suspension from (CLNs), isolated from immunized animals were stimulated ex vivo with 200 μl OVA (20 μg/mL). mRNA was collected after 24 h in culture. Gene expression was normalized to β-actin and fold change was calculated over unstimulated cells for every immunization group. Representative data is mean ± s.e.m of two independent experiments. Statistically significant differences using One-way ANOVA (Kruskal-Wallis multiple comparisons test) *p < 0.05 compared to untreated control group. [file Image_1.tif]
